# GRAPEVNE - Graphical Analytical Pipeline Development Environment for Infectious Diseases

**DOI:** 10.12688/wellcomeopenres.23824.1

**Published:** 2025-05-27

**Authors:** John-Stuart Brittain, Joseph Tsui, Rhys Inward, Bernardo Gutierrez, Gaspary Mwanyika, Houriiyah Tegally, Tuyen Huynh, George Githinji, Sofonias Kifle Tessema, John T. McCrone, Samir Bhatt, Abhishek Dasgupta, Stephen Ratcliffe, Moritz U.G. Kraemer

**Affiliations:** 1 Oxford Research Software Engineering Group, University of Oxford, Oxford, England, UK; 2 Pandemic Sciences Institute, University of Oxford, Oxford, England, UK; 3 Department of Biology, University of Oxford, Oxford, England, UK; 4 Colegio de Ciencias Biológicas y Ambientales, Universidad San Francisco de Quito USFQ, Ecuador, Ecuador; 5 Centre for Epidemic Response and Innovation (CERI), Stellenbosch University, Stellenbosch, Western Cape, South Africa; 6 Oxford University Clinical Research Unit, Ho Chi Minh City, Ho Chi Minh, Vietnam; 7 KEMRI-Wellcome Trust Research Programme, Kilifi, Kenya; 8 Department of Biochemistry and Biotechnology, Pwani University, Kilifi, Kilifi County, Kenya; 9 Africa Centres for Disease Control and Prevention (Africa CDC), Addis Ababa, Ethiopia; 10 Vaccine and Infectious Disease Division, Fred Hutchinson Cancer Center, Seattle, Washington, USA; 11 Department of Public Health, University of Copenhagen, 1352 Copenhagen, Denmark; 12 School of Public Health, 12. MRC Centre for Global Infectious Disease Analysis, Imperial College London, London, UK; 13 Pioneer Centre for Artificial Intelligence, University of Copenhagen, Copenhagen, Denmark; 14 Google Inc., Mountain View, USA

**Keywords:** data science, automated workflows, graphical interface, snakemake, open-source, epidemiology, genomics, outbreaks

## Abstract

The increase in volume and diversity of relevant data on infectious diseases and their drivers provides opportunities to generate new scientific insights that can support ‘real-time’ decision-making in public health across outbreak contexts and enhance pandemic preparedness. However, utilising the wide array of clinical, genomic, epidemiological, and spatial data collected globally is difficult due to differences in data preprocessing, data science capacity, and access to hardware and cloud resources. To facilitate large-scale and routine analyses of infectious disease data at the local level (i.e. without sharing data across borders), we developed GRAPEVNE (Graphical Analytical Pipeline Development Environment), a platform enabling the construction of modular pipelines designed for complex and repetitive data analysis workflows through an intuitive graphical interface.

Built on the *Snakemake* workflow management system, GRAPEVNE streamlines the creation, execution, and sharing of analytical pipelines. Its modular approach already supports a diverse range of scientific applications, including genomic analysis, epidemiological modeling, and large-scale data processing. Each module in GRAPEVNE is a self-contained Snakemake workflow, complete with configurations, scripts, and metadata, enabling interoperability. The platform’s open-source nature ensures ongoing community-driven development and scalability. GRAPEVNE empowers researchers and public health institutions by simplifying complex analytical workflows, fostering data-driven discovery, and enhancing reproducibility in computational research. Its user-driven ecosystem encourages continuous innovation in biomedical and epidemiological research but is applicable beyond that.

Key use-cases include automated phylogenetic analysis of viral sequences, real-time outbreak monitoring, forecasting, and epidemiological data processing. For instance, our dengue virus pipeline demonstrates end-to-end automation from sequence retrieval to phylogeographic inference, leveraging established bioinformatics tools which can be deployed to any geographical context. For more details, see documentation at: https://grapevne.readthedocs.io

## Plan language summary

With the growing amount of data on infectious diseases, researchers have new opportunities to improve public health decisions and pandemic preparedness. However, analyzing this vast and diverse data—spanning clinical records, genomic sequences, epidemiological trends, and geographic information—can be challenging due to differences in data processing methods, technical expertise, and access to computing resources.

To address these challenges, we developed GRAPEVNE, a user-friendly platform that helps researchers build and manage complex data analysis workflows using a visual interface. Built on the Snakemake workflow management system, GRAPEVNE simplifies the process of organizing and running large-scale studies, making it easier to track outbreaks, analyze disease patterns, and process health data efficiently. Its modular approach allows users to customize workflows based on their specific needs, ensuring flexibility and ease of use.

As an open-source platform, GRAPEVNE fosters collaboration and rolling development, supporting a wide range of applications, including genomic analysis, epidemiological modeling, and outbreak monitoring. Researchers can use it for tasks such as studying viral evolution, predicting disease spread, and processing epidemiological data across different geographical contexts. By streamlining data analysis, GRAPEVNE empowers public health institutions and researchers to make data-driven decisions more effectively.

For more details, visit: https://grapevne.readthedocs.io.

## Introduction

Infectious disease outbreaks continue to cause substantial morbidity and mortality. A principle requirement for the control and prevention of outbreaks and pandemics is the systematic and routine collection and analysis of data to reveal the drivers and transmission dynamics, including assessments of optimal control measures effective in reducing spread ^1^. Modern infectious disease analyses increasingly rely on data that spans multiple modalities, including epidemiological, genomic, wastewater, immunological, and spatial to make such assessments ^2– 4^.

Outbreaks and epidemics are multifaceted, with dynamic changes occuring in the host, pathogen and environment. Typically, no single data source can offer a comprehensive understanding of an epidemic or provide sufficient insight for response. To extract the maximum amount of information and insights from disparate data sources they need to be integrated and analysed jointly ^3^. For example, integrating genomic and clinical data during the COVID-19 pandemic in the UK revealed differences in secondary attack rates for new variants and vaccine effectiveness ^5, 6^. Joint analysis of pathogen genomes, international passenger transport volumes, and epidemiological contact tracing data revealed the dynamics and impact of Omicron BA.1 importations ^7^. Existing tools to (semi-)automate analyses often only consider one data type, see for example Nextstrain ^8^ for pathogen genomic analysis and Epiverse for epidemiological analyses ^9^.

During disease outbreaks, analysis of data are often repeated when new information becomes available which increases the burden for data scientists and epidemiologists ^10^. Performing complex analyses with multiple data types, often with varying degrees of data accessibility ^11^, also presents a unique challenge in public health ^12^. Further, due to the potential impact of the analyses performed, the highest standards of reproducibility must be ensured ^13^.

We here present GRAPEVNE (which stands for Graphical Analytical Pipeline Development Environment; github.com/kraemer-lab/GRAPEVNE), a software platform built around the Snakemake workflow management system. GRAPEVNE encourages constructing and collaborating on complex data analysis workflows through an intuitive graphical interface allowing interdisciplinary analyses of data during disease outbreaks. This digital tool is locally installable, is agnostic to the programming language and hardware for execution, and creates workflows that can run without access to the internet (after installation). We demonstrate the utility of GRAPEVNE across two contemporary use cases, i) reconstruction of the global spread of SARS-CoV-2 variants, and ii) Regional and global dengue pathogen genomic analysis.

## Methods

We engaged with ~50 infectious disease modellers, bioinformaticians and genomic epidemiologists to identify key challenges in existing tools for designing and executing analysis workflows during disease outbreaks ( Table 1). During the development, we received iterative feedback from a range of users, including those that would participate in the design of modules (developers with technical and subject expertise) and those who would use pre-existing pipelines with their own data.

**Table 1.** Challenges in the generation and execution of analysis workflows during disease outbreaks and how GRAPEVNE is designed to tackle them.

Process	Challenges	GRAPEVNE functionality
Design	• Necessitates specialist and highly technical knowledge • Requires proficiency in multiple programming languages and domain-specific methodological frameworks/software	• Community-driven catalogue of predesigned modules • Modules provide access to domain-specific tunable parameters with minimal operational knowledge • Ability to deploy workflows in regions where data science capacity is limited
Build	• Complex workflows, frequently dealing with large, noisy, and diverse data sets • Requires a high degree of manual input	• Break-up complex workflows into manageable chunks (hierarchical / nested modules) • Re-use hierarchical modules to rapidly develop new workflows, or adapt existing workflows
Execute	• Highly optimised, specialised to compute infrastructure • Dependencies and version control management	• Relies on *Snakemake* to schedule / execute jobs, which supports many different hardware configurations • *Snakemake* operates sophisticated scheduling heuristics to optimise workflow throughput • Module dependencies are managed through *conda* environments which can be executed in a containerised environment.
Share	• Lack of a centralised repository or ecosystem that enables module sharing	• A repository-based ecosystem of modules to support common functionalities, and permits sharing of bespoke modules
Adapt	• Rewriting code and modules for own project • Limited flexibility for parameter choices	• Workflows present tunable parameters for repeated analysis • Hierarchical modules allow workflows to be deconstructed, adapted and rebuilt with an intuitive no- code interface
Reproduce	• Error-prone • Difficult to reproduce some analyses on own hardware • Comparing results across settings	• Specified workflows will execute the same code each time • Workflows can be packaged / containerised (docker) to avoid local configuration confounds • Workflows can be shared as modules via the repository ecosystem • Due to standardisation of workflows, results can be more easily compared

### Implementation

GRAPEVNE offers a standardized, accessible solution for linking *Snakemake* ^14^ modules with a user-friendly, cross-platform interface supporting Linux, macOS, and Windows. With integrated support for *conda* (conda.io) dependency management, and containerization, GRAPEVNE enables users to test pipelines locally and seamlessly deploy them to High Performance Computing (HPC) or secure cloud environments. By visualizing workflows as graphs and providing easy options to drag-and-drop, reconfigure, and export workflows, GRAPEVNE fosters reproducibility and collaboration, allowing researchers to share, reuse, and adapt workflows within and beyond their teams and applications.

GRAPEVNE is an Electron (electronjs.org) application written in Node.js (nodejs.org) and Python (python.org), with a user-interface underpinned by Typescript (typescriptlang.org) with React (react.dev) components and Redux (redux.js.org/) state management. GRAPEVNE comes bundled with Python 3 and *Snakemake* to facilitate rapid testing (although local installations can be preferred, as required). GRAPEVNE operates by linking modules, which are themselves *Snakemake* workflows that adopt a set of bespoke wrappers designed to provide an interface layer for compatibility and namespace redirection. Workflows can be tested through the user-interface, packaged for execution with *Snakemake*, or containerised for stringent dependency and environment management.

### Operation

GRAPEVNE is available to download for Windows 10 (or later), macOS 11 (or later) and modern Linux distributions. The software can be downloaded from github (github.com/kraemer-lab/GRAPEVNE). To run workflows outside of GRAPEVNE you will require Snakemake 7 (or later; snakemake.github.io), which requires Python 3.7 (or later; python.org), and have the latest version of conda (conda.io) installed.

## Building workflows

**Figure 1.**
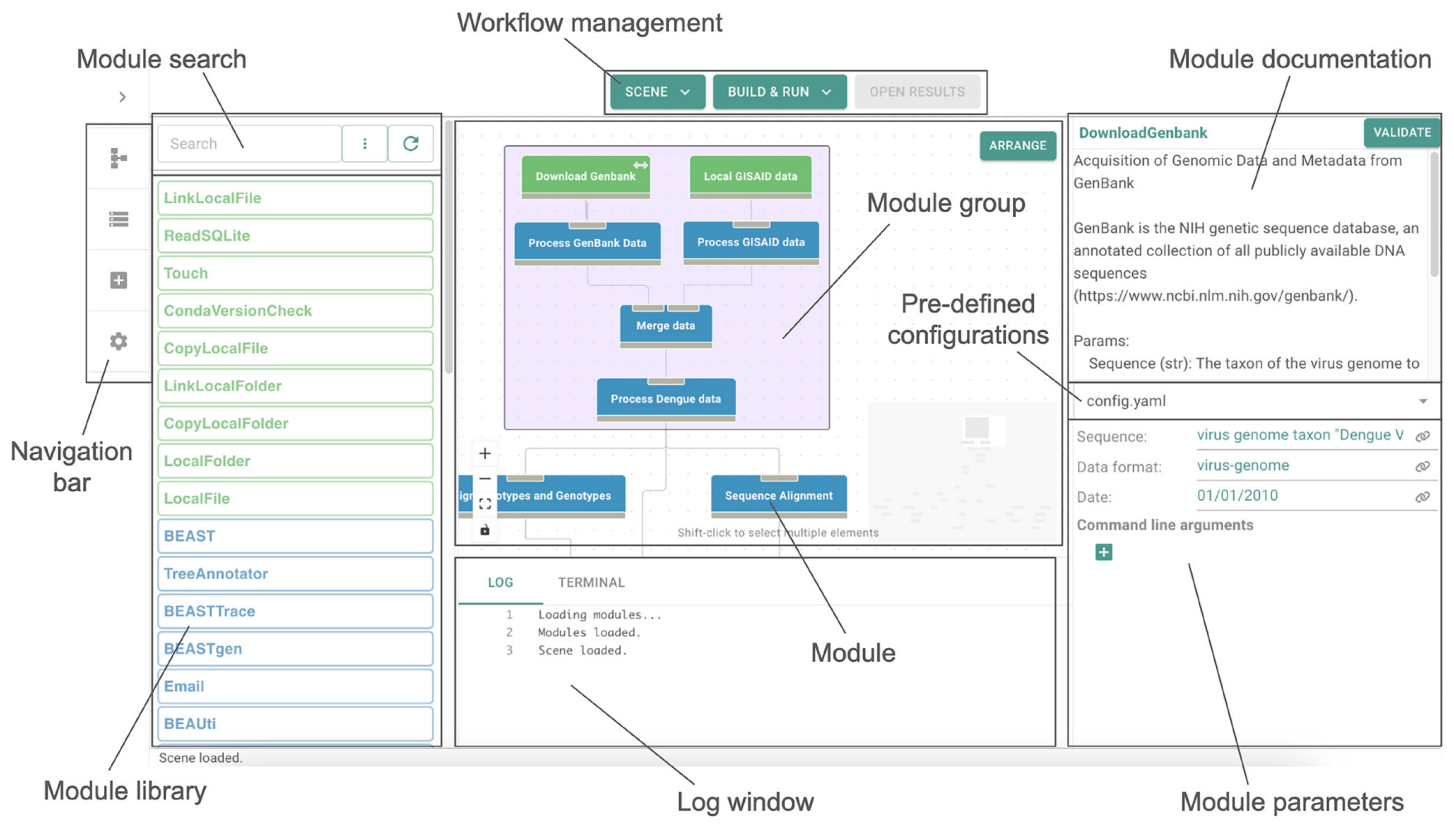
GRAPEVNE interface. (Left) The main canvas shows the workflow under construction with modules and module groups. Selecting a module (‘Download Genbank’ in this example) opens the documentation and configuration panel. Parameter presets can be saved and loaded for convenience. Modules can be browsed from configured repositories via the module library, which includes ‘Repository’, ‘Project’ and free-text search facilities (repositories are configured in the Settings panel, accessible from the Navigation bar). An online catalogue of modules is available via our in-built ‘vneyard’ module browser. Finally, workflows can be tested and packaged for distribution. (Right) Example Snakemake rule making use of grapevne wrappers which manage namespace redirection and compatibility checks. Wrapper functions are highlighted in blue. Several wrappers (such as input, output and params) are represented and can be configured via the GUI. Others, such as script and resource provide support services, such as the provision of payloads, in this case.

The canvas is the main graphical interface where users can construct data processing workflows ( Figure 1). Available modules are displayed in the library panel with filter and search functions. Modules can be dragged into the main canvas area to be incorporated in a workflow. Each module provides its own documentation and can be configured through a set of available parameters. Parameters can be linked between modules to avoid repetitive configuration changes. Modules are hierarchical, allowing different levels of process abstraction to be represented. Hierarchical (nested) modules can be parameter tuned, or expanded permitting structural changes where more extensive modifications are required.

Modules can be stored locally or on remote GitHub repositories. Online access permits a catalogue of modules to be searched and made available to the community, while local repositories offer privacy and/or a development environment. GRAPEVNE allows users to link modules across multiple repositories and has built-in support to keep local repositories synchronised with their remote equivalents. Modules linked from Github will, by default, associate with a commit hash, ensuring version consistency on subsequent runs. These can be updated as newer versions of the module become available. GRAPEVNE provides built-in access to the ‘ *vneyard’*, a searchable set of repositories where common packages and tools can be found, allowing researchers to identify existing modules that might be of use for their research interests.

## Module editor

The Module Editor allows users to create or customize modules by defining a set of requirements, output, and dependencies of the module. This option is particularly useful if the user wants to wrap existing software (either custom written scripts or third-party packages) for use as a GRAPEVNE module. Note that *Snakemake* (and hence GRAPEVNE) is generally agnostic to the language of the scripts provided as these are typically called through the relevant interpreter (e.g. R, Python, bash). Modules can be configured with editable parameters and support payloads for scripts and/or additional resources. Software dependencies are supported via *conda*. The module editor allows the user to 1) easily wrap existing software/scripts that are not yet available in the GRAPEVNE ecosystem, and 2) permit rapid development and incorporation of custom scripts into new or existing pipelines.

Internally, modules are *Snakemake* workflows where the Snakefile and configuration files follow specific rules. These details are typically never exposed to the user since the Module Editor allows a wide array of software to be encapsulated for use through the GUI. However, where modules require custom elements or refined control, the user may interact with their associated Snakefile directly through our GRAPEVNE wrapper set which provides compatibility checks and namespace redirection (functionality is detailed in our documentation: https://grapevne.readthedocs.io).

## Execution manager

Once a workflow is built, it can be tested (executed locally through the GUI) or exported as a self-contained *Snakemake* workflow. During testing, logs are displayed in real-time through the GUI, which streamlines debugging and workflow optimization. Meanwhile, the exported workflow does not require GRAPEVNE to be present on the host system and can be executed entirely through *Snakemake*, which manages each modules’ dependencies through *conda*. Note that we also support packaging the workflow in a container, wherein only the container manager is required for execution. In this case, *Snakemake* and all module dependencies are set up within the container, which can be prepared ahead of execution, while also maximising reproducibility across different systems. In either case, the workflows are ultimately managed by *Snakemake*, a widely adopted tool which ensures scalability and fault tolerance. As such, the workflow can be executed on the wide variety of compute environments supported by *Snakemake*, including local compute, cloud-based environments, and HPC clusters. The workflows produce execution logs, and GRAPEVNE can easily be configured to send email notifications to inform users of any workflow status updates, including when the job is finished and whether it succeeded (or failed).

## Use cases

### Global dispersal of SARS-CoV-2 Variants of Concern

During the COVID-19 pandemic, there were multiple waves of infections driven by the emergence of new virus variants resulting from continued antigenic evolution. These variants have been classified by the World Health Organization (WHO) as Variants of Concern (VOCs) on the basis that they exhibit increased transmissibility and/or immune escape and therefore should be prioritized for global monitoring, research, and vaccine/therapeutic development ^15^. Understanding the global dispersal dynamics of these VOCs in the context of the continuously shifting landscape of public health interventions, population immunity, and human behaviours and mobility is critical for informing the design of effective control measures. Numerous studies have explored various aspects of this question, and it remains an active area of research as researchers continue to draw valuable insights from the vast amount of data generated during the pandemic.

As a proof-of-concept and to facilitate ongoing research efforts, we reimplemented within GRAPEVNE the analysis performed by Tegally *et al.* - a study investigating how the global dispersal patterns of multiple VOCs (specifically Alpha, Beta, Gamma, Delta, and Omicron BA.1 & BA.2) were shaped by the air traffic network ^16^. This particular study was selected for three primary reasons: (1) to reconstruct the spatiotemporal spread of each VOC, the authors employed a phylogeographic approach which requires the coordination of multiple third-party software packages and custom scripts written in different programming languages, (2) to ensure comparability, the same analysis was performed for each VOC separately (and further replicated across 10 random subsets of sequences), and (3) a large number of sequences (~20,000) were analysed for each VOC, rendering the manual execution of the analysis both time-consuming and error-prone.

**Figure 2.**
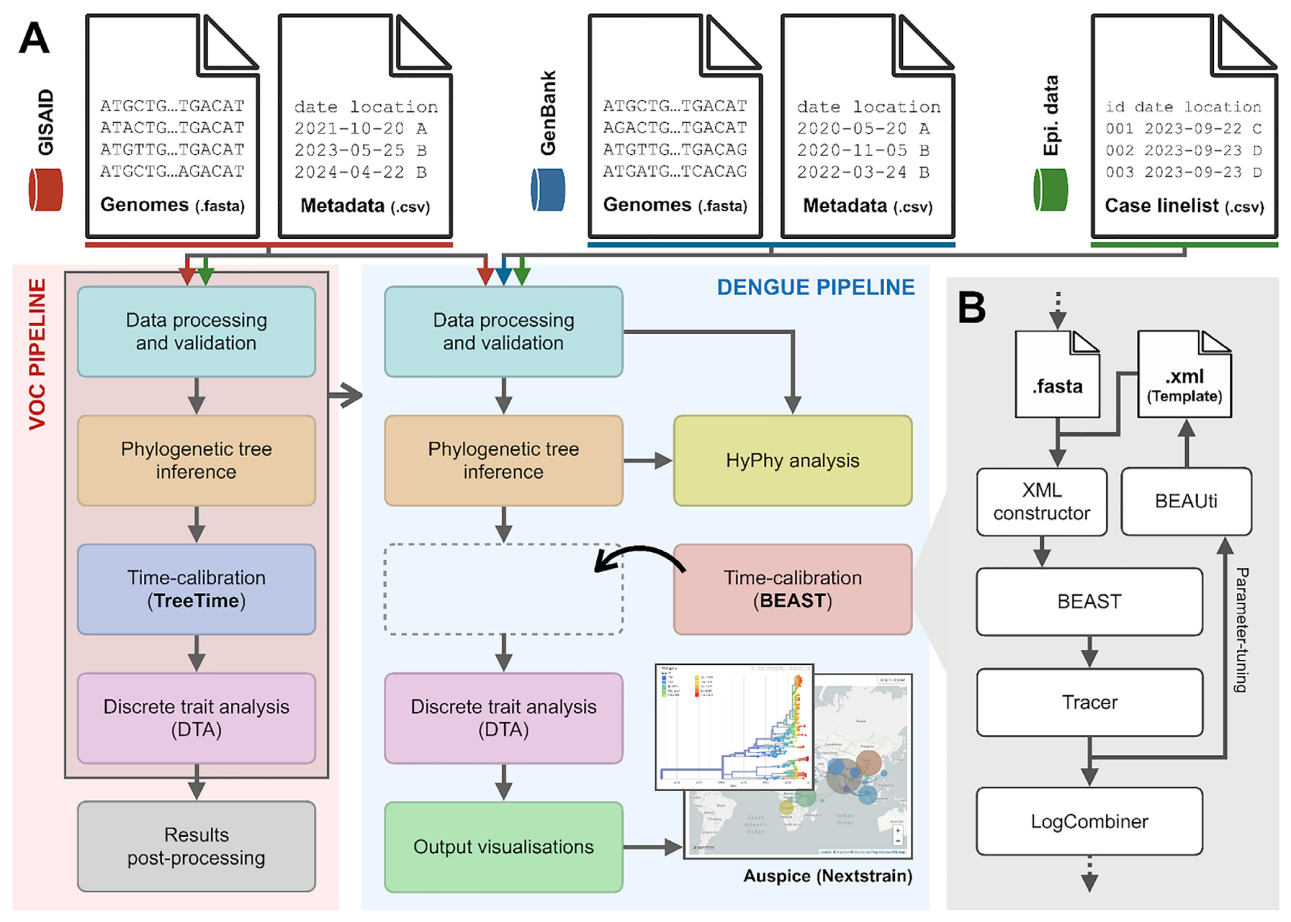
Design and implementation of analytical pipelines for reconstructing the spread of SARS-CoV-2 Variants of Concerns (VOCs) and Dengue virus. ( **A**) The red panel illustrates the high-level structure of the SARS-CoV-2 VOC pipeline, integrating genomic data from GISAID ^28^ (red arrow) and epidemiological data from other sources (e.g., case data from OWID ( https://ourworldindata.org/); green arrow) to infer the historical dispersal patterns of the virus at a global scale. This pipeline serves as a template (grey box) for the Dengue pipeline in the blue panel, with three key modifications: (i) the time-calibration module based on TreeTime ^19^ is replaced by an equivalent module based on BEAST instead, (ii) an additional module is added to perform evolutionary hypothesis testing using HyPhy ^32^, and (iii) an additional module is added to visualize output from the discrete trait analysis using auspice ^8^. ( **B**) An expanded view of lower-level modules nested within the time-calibration module using BEAST ^30^. A FASTA file containing pathogen genomes is used as input to generate an XML file, following the configurations as specified in an XML template generated by the user through a graphic user-interface application known as BEAUti. The XML file is then used as input by BEAST to perform Markov chain Monte Carlo (MCMC) sampling. Intermediate output is visualized and assessed for convergence using Tracer. The user then has the option to either continue running the analysis and proceed with further downstream analyses (e.g., generating the maximum clade credibility (MCC) tree using LogCombiner), or to modify the XML (e.g., tuning parameters associated with prior distributions within BEAUti) and rerun the BEAST analysis in an iterative fashion.

The analysis workflow implemented in GRAPEVNE consists of the following steps: (1) sequence alignment using NextAlign ^8^, (2) downsampling of sequences in proportion to case number per country per week using a subsampler developed by Brito *et al.* ^17^, (3) phylogenetic tree inference using FastTree ^18^, (4) removal of temporal outliers via manual inspection, (5) time-calibration using TreeTime ^19^, (6) discrete-trait analysis (DTA) ^20^ using TreeTime ^21^, and finally (7) the extraction of inferred viral movements from the DTA output. For a given VOC under investigation, the user has to provide as input a FASTA file containing the sequences to be considered for inclusion in the analysis, and a CSV file containing weekly variant-specific case data for each country with available genomes. To automate the replication across random subsets of sequences, users can additionally specify a list of random seeds which are then used to randomly sample sequences from the input genomic dataset. The modular design of the pipeline allows each step to be run independently (provided that the required input files are available) and the output inspected before proceeding to the next step. This represents an advantage particularly for phylodynamic and phylogeographic analyses, where it is common to iteratively refine the analysis by adjusting parameters (e.g., different prior distributions for evolutionary rate) or switching software packages with differing implementations and underlying assumptions (e.g., IQ-TREE ^22^ instead of FastTree for smaller datasets and model testing) based on results from previous steps. ( Figure 2)

### Dengue genomic epidemiological pipeline

Dengue virus (DENV) is the fastest expanding vector-borne pathogen with an estimated 2.5 billion people at risk of infection ^23^. DENV exists as four genetically and antigenically distinct serotypes (DENV-1 to DENV-4), each further subdivided into multiple genotypes and sublineages that differ in their geographical distribution and expansion patterns ^24^. Over the past five decades, the virus has broadened its geographic range, spreading into regions previously considered non-endemic, including southern Europe, China, Brazil, and the United States, a trend driven by increased global connectivity, human mobility, climate change, and urbanisation ^25– 27^.

The potential for large outbreaks has been shown to increase when new genotypes/serotypes spread to new areas, necessitating tracking of the spatio-temporal dynamics of specific DENV lineages. To address this need, we developed and deployed a modular analysis pipeline within GRAPEVNE that can be run end-to-end, executed selectively (as individual modules or module collections/branches), or expanded upon creating new branches. The pipeline’s first branch harmonises and merges DENV pathogen genomic data from public repositories GISAID ^28^, GenBank ^29^ and private local datasets, cleaning the datasets and removing duplicates, to create comprehensive datasets encompassing all four DENV serotypes. Subsequently, a processing branch assigns genotype and lineage, aligns sequences, and separates them into E-genes or whole genomes. Further downstream, an analysis preparation branch subsamples data using proportional or weighted approaches ( https://github.com/kraemer-lab/vneyard), before further branching into three analytic pathways: maximum likelihood approaches (IQ-TREE-2 ^22^, TreeTime ^19^, Nextstrain ^8^), Bayesian (BEAST, Beauti ^30^, Fertree ^31^), and hypothesis testing (HyPhy ^32^). Each branch includes custom scripts for analyses, for example, extracting introductions from phylogenetic trees annotated with branch locations, and visualisations (including maps showing the spatio-temporal spread of each serotype and major genotype), for ease of interpretation. Researchers have the option to incorporate only GISAID/GenBank data or/and locally generated datasets, drag-and-drop modules and branches to fit specific project needs or to answer specific scientific questions, and quickly add new or modified branches. The pipeline itself can be shared without revealing any underlying data, fostering rapid analytics while preserving data confidentiality where necessary. This is especially useful in scenarios where standardized analyses have to be performed and reproduced across multiple locations without sharing potentially sensitive genomic data. Access conditions when retrieving publicly available data must comply with the requirements of individual repositories; for this pipeline, GISAID ^28^ data access and usage requires the users to provide their individual credentials and to comply with the terms and conditions outlined on the platform.

Compared to existing tools such as Nextstrain ^8^ (which also relies on *Snakemake*), GRAPEVNE’s visual interface makes it easier to modify, add, or remove modules, offering a higher degree of flexibility and customisation while preserving some of the key functionalities of other tools. Its drag-and-drop functionality removes the technical barriers of entry. GRAPEVNE’s visual workflow also clarifies each module’s requirements, which parameters have been and need to be specified, necessary input files, and the resulting outputs, while clearly illustrating how modules connect both upstream and downstream. This visual approach greatly enhances pipeline trackability, making it far easier to troubleshoot issues and minimising issues that might arise when parsing and sharing complex Snakefiles.

When working with heterogeneous datasets (e.g., DENV) or large datasets (e.g., SARS-CoV-2), researchers often split data into genotypes or lineages. In turn, each subset demands its own cleaning, processing, and analysis steps, dramatically increasing the time and effort required. Without a workflow manager, these tasks become time-consuming and prone to error. By automating and streamlining the process, GRAPEVNE reduces repetitive workloads, mitigates human error, and ensures scalability for complex genomic analyses, especially when they have to be re-performed as data gets updated.

## Discussion

Infectious diseases, seasonal and emerging, are continuing to impact societies globally. While integrated disease surveillance has greatly advanced the ability to detect, monitor, assess and forecast disease spread, systematic and scalable approaches at a global level are still lacking. We here built a platform that fosters interdisciplinary research by enabling experts with different subject and technical expertise to collaboratively design complex analytical workflows, with a focus on streamlining joint analyses of multi-modal data for time-critical disease outbreak investigations.

GRAPEVNE was built to encourage contributions from the scientific community to organically evolve to accommodate the needs of data scientists and modellers working towards more scalable and robust approaches to infectious disease modelling. Through its platform-agnostic approach, GRAPEVNE is designed to be robust to varying degrees of local needs, capacity, and access to computational infrastructures (execution on cloud, local machine or HPC). Sharing workflows between parties and in any programming language aids collaborations across fields with different conventions. For example, the epidemiological modelling community relies heavily on R while machine learning researchers primarily programme in Python - as a result, fostering interdisciplinary collaboration between these two communities has been challenging in the past.

Natively, *Snakemake* operates on a dependency-driven directed acyclic graph (DAG) model, where rules define input-output transformations, and execution order is dictated by dependencies rather than strict sequencing. Its Python-based, rule-centric design makes it particularly accessible for local or cluster-based workflows, with automatic resource management. The integration with Python offers substantial flexibility for complex logic, although it does require users to be comfortable with scripting. The GRAPEVNE package adds significant flexibility to *Snakemake* workflows by introducing wrapper functions around input and output files, allowing users to rearrange module order dynamically which is common when designing and reusing modules for pipelines. This capability simplifies prototyping and iterative adjustments, which are common in bioinformatics, epidemiological modelling and data science, without altering core dependencies. GRAPEVNE’s modular hierarchy, with sub-module nesting, enhances organization and scalability, facilitating the management of complex pipelines. By providing a flexible yet structured framework, GRAPEVNE allows researchers to adapt workflows for diverse experimental designs or data processing steps with minimal restructuring.

While we have built upon Snakemake, there are of course many other workflow managers available. One popular choice in genomics is Nextflow which employs a dataflow model where processes are linked by data channels, making it well-suited for cloud-native and high-performance computing (HPC) environments. With workflows specified in Groovy, Nextflow emphasizes a reactive, event-driven execution style, with data availability triggering process execution. In contrast, Snakemake specifies workflows in Python, a language that has become ubiquitous in data science and data analysis due to its high degree of flexibility and shallow learning curve. In addition there are several existing no-code solutions available for workflow construction, such as Data-flo (data-flo.io), which offers a streamlined, visual approach to data integration. However, while GRAPEVNE is accessible as a no-code platform, it also provides a high degree of flexibility to customize modules through its dynamic wrapper system. This empowers the user to customize modules in Python, modify their interactions, adjust dependencies, and integrate custom logic while maintaining a structured, modular workflow, ensuring both high-level usability and deep configurability for advanced users. GRAPEVNE thus fills a unique niche, offering a low barrier to entry via a no-code solution with community focussed module repositories, the flexibility of Python for more advanced users, and a workflow model underpinned by a dependency-driven, yet modular approach.

Building on the lessons learned from the COVID-19 pandemic, we hope that this platform will improve the efficiency of processing, analysis, and sharing of large genomic datasets accelerating our timely response and enhancing epidemic preparedness.

We encourage contributions from the wider data science, bioinformatics, and epidemiological community, translating their packages, pipelines, and tools into GRAPEVNE modules (see a searchable list of current modules here: https://kraemer-lab.github.io/vneyard/). GRAPEVNE is a step forward in using data science to improve infectious disease related computational analyses to unlock new insights ^33, 34^.

## Ethics and consent

Ethical approval and consent were not required.

## Data Availability

Data for the *Global Dispersal of SARS-CoV-2 Variants of Concern* use case is available from the GISAID ( https://gisaid.org/) EpiCov database (GISAID: EPI_SET_230221dt). Data for the *Dengue Genomic Epidemiological Pipeline* use case is available from the GISAID EpiArbo database and from the GenBank database hosted by the National Center for Biotechnology Information (NCBI). The GISAID EpiArbo dataset comprised all sequences with a Human host possessing a collection date later than 2000-01-01 and a submission date of up to 2024-11-18, inclusive. GenBank data is downloaded automatically as part of the GRAPEVNE workflow. GISAID access is facilitated through the Database Access Agreement between individual users and GISAID. It is the responsibility of individual users to comply with the terms of the Database Access Agreement.

## Software availability

Source code for GRAPEVNE is available from ( https://github.com/kraemer-lab/GRAPEVNE) under MIT license ( https://mit-license.org). Archived source code is available from https://doi.org/10.5281/zenodo.15358021 ^35^.
