# Supplementary material for: GRAPEVNE - Graphical Analytical Pipeline Development Environment for Infectious Diseases
Source: Wellcome Open Res. 2025 May 27;10:279. [Version 1] doi: 10.12688/wellcomeopenres.23824.1 (PMC12205304; doi:10.12688/wellcomeopenres.23824.1)
Supplement: Peer review history

# Reviewer report: Reviewer response for version 1

**DOI:** 10.21956/wellcomeopenres.26280.r126316

**Published:** 2025-06-28

**Authors:** Kayode Oshinubi

**Affiliations:** 1 Northern Arizona University, Flagstaff, Arizona, USA

This article provides a thorough explanation and usage of a new epidemiological data analysis tool. I find this article to be interesting and I do not have review comments for the authors but rather commend them for a job well done. I appreciate the author's openness and for providing the link for the package. I found the package easy to use and it will add value to this research endeavor across the globe.

Are the conclusions about the tool and its performance adequately supported by the findings presented in the article?

Yes

Is the rationale for developing the new software tool clearly explained?

Yes

Is the description of the software tool technically sound?

Yes

Are sufficient details of the code, methods and analysis (if applicable) provided to allow replication of the software development and its use by others?

Yes

Is sufficient information provided to allow interpretation of the expected output datasets and any results generated using the tool?

Yes

Reviewer Expertise:

computational epidemiology, infectious disease modeling, data modeling, biostatistics

I confirm that I have read this submission and believe that I have an appropriate level of expertise to confirm that it is of an acceptable scientific standard.

# Reviewer report: Reviewer response for version 1

**DOI:** 10.21956/wellcomeopenres.26280.r124600

**Published:** 2025-06-16

**Authors:** Anderson F Brito

**Affiliations:** 1 Instituto Todos pela Saúde (ITpS), São Paulo, Brazil

The manuscript presents GRAPEVNE, a modular, no-code, open-source platform for constructing and executing analytical workflows for infectious disease data analysis. Built on Snakemake and complementary technologies, it provides a graphical interface accessible to users with varying levels of technical expertise. Its utility is demonstrated through two case studies: SARS-CoV-2 variant tracking and dengue virus surveillance. Although I was not able to install and test the tool (I've encountered compatibility issues with MacOS Ventura), the tool appears to be a flexible and scalable solution, allowing the integration of many existing tools, a key challenge for reproducibility and usability during outbreaks.

 To enhance clarity and usability, I suggest the following improvements:

- Provide direct access to replication materials by including explicit links to walkthroughs and datasets for at least one case study: I've found such resource on grapevne's website/repositories. This would help readers reproduce the workflows and better understand how to build their own pipelines.
- Clarify the process for validating community-contributed modules. While the manuscript mentions community-driven sharing, it does not specify whether there is any mechanism for testing, review, or version control to ensure module quality and compatibility.

Are the conclusions about the tool and its performance adequately supported by the findings presented in the article?

Yes

Is the rationale for developing the new software tool clearly explained?

Yes

Is the description of the software tool technically sound?

Yes

Are sufficient details of the code, methods and analysis (if applicable) provided to allow replication of the software development and its use by others?

Partly

Is sufficient information provided to allow interpretation of the expected output datasets and any results generated using the tool?

Partly

Reviewer Expertise:

Virology; Bioinformatics; Public Health; Genomics

I confirm that I have read this submission and believe that I have an appropriate level of expertise to confirm that it is of an acceptable scientific standard.
